# CNS Genes Implicated in Relapse

**DOI:** 10.4137/sart.s1042

**Published:** 2008-09-25

**Authors:** Kara L Kuntz-Melcavage, Willard M Freeman, Kent E Vrana

**Affiliations:** Department of Pharmacology, Pennsylvania State University College of Medicine, R130, 500 University Drive, Hershey, PA 17033, U.S.A.

**Keywords:** gene expression, drug relapse, epigenetics, gene intervention

## Abstract

Drug abuse is a condition that impacts not only the individual drug user, but society as a whole. Although prevention of initial drug use is the most effective way to prevent addiction, avoiding relapse is a crucial component of drug addiction recovery. Recent studies suggest that there is a set of genes whose expression is robustly and stably altered following drug use and ensuing abstinence. Such stable changes in gene expression correlate with ultrastructural changes in brain as well as alterations in behavior. As persistent molecular changes, these genes may provide targets for the development of therapeutics. Developing a list of well-characterized candidate genes and examining the effect of manipulating these genes will contribute to the ultimate goal of developing effective treatments to prevent relapse to drug use.

## Introduction

Relapse to drug use is a major barrier to overcoming addiction (Stewart, 2008; Leshner, 1996). Identifying changes in brain gene expression that underlie relapse liability will ultimately lead to a better understanding of what drives recovering addicts to relapse to drug use. Such an understanding will subsequently provide molecular targets on which pharmacological interventions to prevent relapse can be focused. Drug use alters the individual on multiple levels: behavior, neurochemistry, electrophysiology, and microanatomy, but underlying all these changes are gene and protein expression modifications (for review see Nestler, 2001 and Falcon and McClung, 2008). This review briefly describes several behavioral models of relapse, identifies a list of genes that are commonly found to be involved in relapse, and highlights epigenetic studies that are delving further into the role of genomic contributors to relapse. Studies that combine behavioral and molecular techniques to examine the relevance of certain genes to relapse are discussed, as are future directions of study to advance development of treatments for relapse.

## Models of Relapse

**Figure 1:**
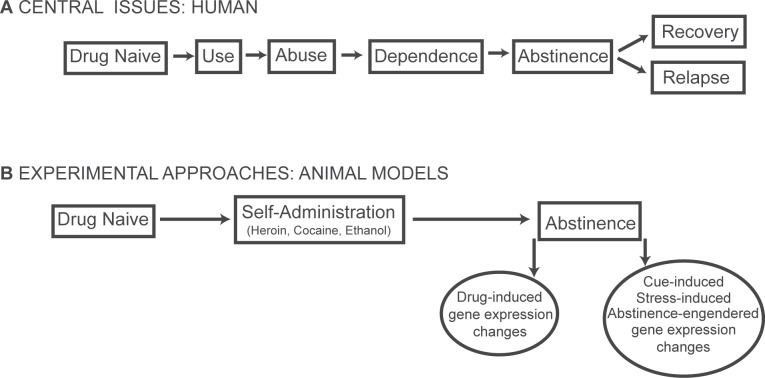
(**A**) Human drug use ends in either successful sustained abstinence or relapse. (**B**) Drug relapse is modeled in animals by cue-induced or stress-induced resumption of drug self-administration following an abstinent period. Categories of gene changes following drug use are depicted in ovals.

Human relapse to drug use is an overarching problem addressed by studies of addictive drugs. For humans, relapse to drug use can occur even after substantial treatment for addiction and prolonged abstinence (DeJong, 1994; O’ Brien, 2003). The difficulty in modeling relapse to drug use in animals has presented a challenge to researchers interested in the genomic underpinnings of addiction. Clearly, an effective model of relapse will replicate the human experience of drug use and relapse (Fig. 1A). Currently, a few behavioral relapse models exist, providing an effective way to study relapse in the controlled laboratory environment. Although these models differ slightly from one another, all adhere to the central experimental approach of drug self-administration followed by a period of abstinence (Fig. 1B).

Incubation is the most recently recognized model of relapse. Occurring in response to both stimulants and opiates, incubation is described as a time-dependent increase in cue-induced operant responding after withdrawal from self-administration (Grimm et al. 2001; Lu et al. 2006). Incubation occurs following self-administration of cocaine (Freeman et al. 2008), methamphetamine (Shepard et al. 2004), and heroin (Shalev et al. 2001; Kuntz et al. 2008a); therefore, it may represent a generalizable phenomenon applicable across addictive drugs.

For both animals and humans, reinstatement of drug seeking behavior can be triggered by re-exposure to the previously self-administered drug, the context in which it was taken, or specific environmental cues. In the animal model of extinction-reinstatement, drug seeking behavior is extinguished by multiple sessions during which no drug is received following responses on a previously drug-paired operant. In this paradigm, the drug reinforcer is disassociated from the behavior. Following extinction of the drug-seeking behavior, a stimulus such as an injection of the drug, re-exposure to the environment in which drug was received, or a stressful event can trigger resumption of the drug-seeking behavior. Extinction-reinstatement is known to occur for both cocaine-seeking behavior and heroin-seeking behavior (Crombag et al. 2008; Rogers et al. 2008). Extinction-reinstatement has been a widely used animal model for studying relapse, although the applicability of this model to human drug relapse has been debated (Katz and Higgins, 2003; Epstein et al. 2006). Extinction of drug-seeking is a crucial component of the extinction-reinstatement model in animals; however an analogous situation does not occur in humans. Therefore, the experience of animals that undergo the extinction-reinstatement procedure is quite different from that of human relapsing drug users.

Sensitization to a drug involves an increased drug response to the same amount of a drug following its repeated administration (Feldman et al. 1997; Kalivas et al. 1998a; Kalivas et al. 1998b; Smith et al. 2008). While not a faithful behavioral model of relapse, sensitization provides a highly visible demonstration of how responses to drugs remain altered following a period of abstinence from drug use (Vezina, 2007). Although sensitization occurs for a variety of drugs, it is best expressed following administration of psychomotor stimulants. In psychomotor sensitization, an animal receives injections of a psychomotor stimulant (e.g. cocaine) for a period of time and eventually becomes hyperresponsive to an injection of the stimulant. Both incubation and sensitization provide models of enduring behavioral changes that persist past cessation of drug administration.

Human studies of relapse can provide important insights but are confounded by numerous variables, such as polydrug use, different drug doses, and variations in lifestyle. Because of the confounding variables presented by human studies, animal relapse models provide the opportunity to study relapse in a controlled environment where animals can be manipulated by genetic, behavioral, and pharmacological methods.

## Genes Implicated in Relapse

**Figure 2:**
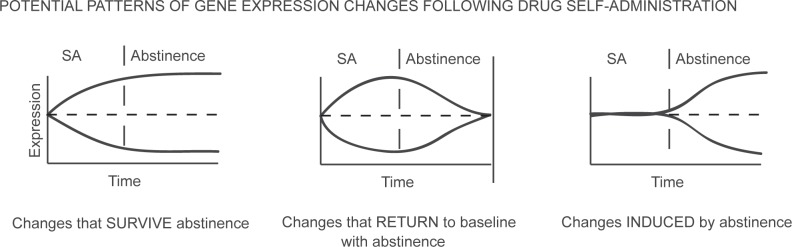
Changes in gene expression can by classified into three broad groups.

**Figure 3:**
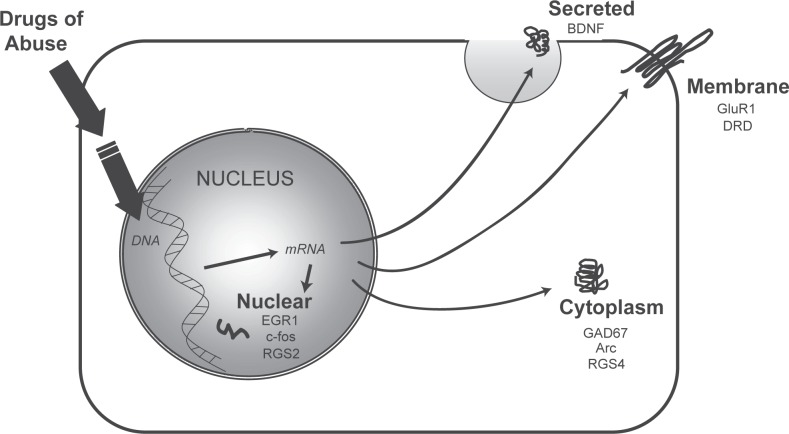
Drug abuse affects transcription of genes that ultimately have intranuclear, cytoplasmic, and plasma membrane actions.

The animal models of relapse described above have been used to identify genes that are significantly altered following drug use and abstinence. The progression from initial drug use to dependence and addiction is characterized by multiple behavioral and physiological changes. Alterations in gene expression are believed to underlie these broader changes and have been demonstrated to alter behavior (Nestler et al. 2001; McClung and Nestler, 2003). Following drug use and abstinence, genomic changes can be divided into three broad categories (Fig. 2). Expression of particular genes can be increased or decreased during the period of drug-administration and arrive at a new steady-state of expression that is maintained throughout a period of drug abstinence. In this case, the physiologic condition produced by the new levels of gene expression can drive a former drug-user to relapse. In a second scenario, drug-administration again induces a change in gene transcript expression. However, as the time of abstinence from the drug increases, transcripts return to their pre-drug level of expression. These genes are less likely to be responsible for long-term relapse to drug use, but they may be necessary initial factors for more enduring secondary expression changes. A third group of genomic changes appear during the abstinent period following drug use. In animal studies, it is known that some of these changes result from re-exposure to a previously drug-paired context. In each of the described scenarios, drug use results in a homeostatic imbalance that is ultimately manifested in an individual’s behavior. Given the multitude of genes present within a genome, when examining a particular behavior (such as relapse), it is useful to focus on a subset of genes believed to be involved in that behavior. The number of genes believed to be associated with drug use exceeds 100 (Worst et al. 2005; Kreek et al. 2005; Hemby, 2006; Yano and Steiner, 2007). However, the majority of these gene expression changes are present only during drug administration (Fig. 2, middle panel). Therefore, far fewer genes remain altered following a period of abstinence. A list of several genes that have been found to be altered in a variety of relapse models is provided in Table 1. Many of these genes are transcription factors, while others are involved in dopamine and G-protein signaling. The external factor of drug abuse is able to induce intranuclear changes that eventually affect entire cells, neighboring cells, and ultimately the physiology of an entire organism (Fig. 3). Genes identified in this article range from transcription factors that exist within the nucleus to genes encoding proteins that act intracellularly to those found within the plasma membrane to secreted factors.

One well-known addiction-related gene is early growth response 1 (EGR1, a.k.a., zif 268, NGFI-A, and krox 24). We have recently shown EGR1 gene expression to be increased in the medial prefrontal cortex (mPFC) of rats that exhibit incubation of heroin-seeking behavior following 14 days of abstinence from heroin self-administration (Kuntz et al. 2008b) and EGR1 has been repeatedly implicated in drug relapse by other laboratories (For recent examples, Schmidt et al. 2005; Lee et al. 2005; Covington et al. 2005, although a wealth of additional studies exist). In an illustration of how specific genes are affected in relapse models, either increased or decreased in expression depending on the specific drug previously used, expression of EGR1 is decreased in the mPFC of rats that have been abstinent from cocaine self-administration for either 1, 10, or 100 days (Freeman et al. 2008), while it is increased following abstinence from heroin (Kuntz et al. 2008b). The divergent directions of changes between the heroin and cocaine studies raises the question of whether these differences can be attributed to the depressive verses stimulant properties of heroin and cocaine, respectively, or variations in the psychodependence and physical dependence induced by different drugs. However, another study of relapse to cocaine seeking has reported mPFC EGR1 gene expression to be significantly increased rather than decreased following cocaine abstinence and contextual re-exposure. In this study, genes that were upregulated upon exposure to an environment in which cocaine was previously administered are not upregulated when rats are re-exposed to an environment in which cocaine was not previously available (Hearing et al. 2008a). Hearing’s study included contextual re-exposure while the samples in Freeman’s study were from rats that had not experienced contextual re-exposure. Re-exposure to a drug-related context is known to impact gene expression (Badiani et al. 1998; Ferguson et al. 2003; Badiani and Robinson, 2004) and offers a plausible explanation for the between-study differences. Variations in the cocaine dose (0.6 mg/kg verses 1.5 mg/kg), operant training method (discrete trial model versus fixed ratio), duration of training sessions (6 h verses 2 h), and RNA quantitation techniques are all possible reasons for the variations in results. Region-specific upregulation of EGR1 has also been observed in rats that experience an amphetamine sensitization challenge (Guitart-Masip et al. 2008). Subregions of the amygdala and striatum varyingly display increases or decreases in EGR1 gene expression, with the direction of change varying depending on the inbred characteristics of the strain and the specific brain region. For most of the subregions examined, EGR1 expression increased in rats that experienced an amphetamine challenge.

Brain-derived neurotrophic factor (BDNF) has been implicated in relapse to drug-seeking behavior. An upstream regulator of the immediate early genes Fos and Arc, BDNF promotes cocaine-taking and relapse behavior in rats (Graham et al. 2007). Interestingly, in the ventral tegmental area (VTA) BDNF protein levels have been found to be increased 10–15 days following withdrawal from cocaine injections, but not 1 day following withdrawal (Pu et al. 2006). Acute, but not chronic, cocaine administration increases striatal BDNF mRNA in rats (Liu et al. 2008). Thus, it is possible that an initial rise in BDNF mRNA levels is followed by subsequent increase in protein levels before the BDNF gene expression returns to a baseline. Because BDNF controls expression of other genes that are implicated in relapse, such as Fos and Arc, BDNF may be a crucial “switch” that activates numerous relapse-related intracellular cascades. Following cocaine withdrawal, levels of BDNF mRNA are reportedly increased in the hippocampus (Filip et al. 2006). Gene expression levels of trkB, the receptor for BDNF, were decreased in this study. Perhaps the decrease in receptors is indicative of a physiological response to counteract the increased levels of BDNF expression. Taken together, these findings suggest that changes in BDNF gene expression are both time and contingency-dependent. The suggested importance of BDNF to drug abuse has prompted studies investigating human polymorphisms of the BDNF gene; however, currently only modest associations between BDNF gene variants and substance abusers have been identified (Itoh et al. 2005; Liu et al. 2005). Levels of BDNF mRNA are increased following a single injection with cocaine (Le et al. 2005), as are levels of the dopamine D3 receptor (DRD3). BDNF is known to control DRD3 mRNA expression (Guillin et al. 2001), and this finding of concurrent increases in expression of these genes provides support for an interaction between these two genes. A reasonable hypothesis is that increased BDNF gene expression levels are responsible for increased DRD3 gene expression levels.

Increased dopamine signaling is an essential component of drug abuse (DiChiara and Bassareo, 2007). Five different subtypes of receptors exist for dopamine: D1, D2, D3, D4, and D5. Expression levels of the genes encoding these receptors have been examined in both human and animal studies of relapse, with expression of specific DRD alleles being prevalent in humans who exhibit addictions to smoking and gambling (Comings et al. 1997). The precise role of DRD and dopamine in addictive behaviors remains to be elucidated, but the generalization of DRD across a range of addictive behaviors suggests this gene may be relevant to addiction beyond the specific area of drug abuse. The mesolimbic dopaminergic system, often referred to as the reward pathway, is important for the appetitive drive to engage in a variety of goal-directed behaviors, regardless of whether they involve drugs (Alacro et al. 2007). Increasing DRD2 expression in the nucleus accumbens reduces ethanol consumption in rats (Thanos et al. 2004), while DRD2 knock-out mice that are treated with DRD2 vectors suggest that DRD2 levels play a complex role in regulating the amount of alcohol consumed (Thanos et al. 2005). Human heroin abusers possessing a polymorphism in the DRD4 or DRD2 genes report significantly higher levels of heroin craving than counterparts who do not possess either of these polymorphisms (Li et al. 2006; Shao et al. 2006). Polymorphisms in the DRD2 and DRD4 genes are believed to be predictive of how successful a person will be at smoking cessasion (David et al. 2007; David et al. 2008). Although these studies examine polymorphisms rather than changes in gene expression, the implication is the same as with expression studies: aberrances in dopamine receptor genes are suspected to be involved in drug use.

Regulators of G-protein signaling are intracellular proteins that terminate G-protein signaling by accelerating GTPase activity (Krumins et al. 2004; Xie and Palmer, 2005; Abramow-Newerly et al. 2006). Several genes exist in this protein family, and while the full extent of their involvement in intracellular functions remains to be elucidated, signaling by dopamine receptors and opioid receptors is known to involve RGS molecules. Messenger RNA for RGS2 and RGS4 is increased following opiate withdrawal (Gold et al. 2003). RGS4 mRNA in the prefrontal cortex and dorsolateral striatum is significantly decreased 21 days following either contingent or non-contingent cocaine administration. Interestingly, in both brain regions, re-exposure to the context in which cocaine was administered prior to the abstinence period restored RGS4 gene expression levels (Schwendt et al. 2007). Acute morphine increases RGS9 protein, while chronic exposure decreases the protein levels (Zachariou et al. 2003). RGS expression can be affected by dopamine signaling, and expression of RGS2, RGS4, and RGS9 are significantly decreased in dopamine D1 receptor knock-out mice (Stanwood et al. 2006). Cocaine induces an increase in RGS4 gene expression through D1 receptors (Zhang et al. 2005). Acute amphetamine decreases RGS4 mRNA expression in rat forebrain (Schwendt et al. 2006). Again, induction or reduction of mRNA expression is seen for RGS4 depending on whether the drug being studied is an opiate or a stimulant. Unfortunately, varibles in drug-taking schedules and abstinent periods necessitate the design of a specific study to assess whether the different directions of change in RGS4 gene expression are solely the result of the drugs studied.

**Figure 4:**
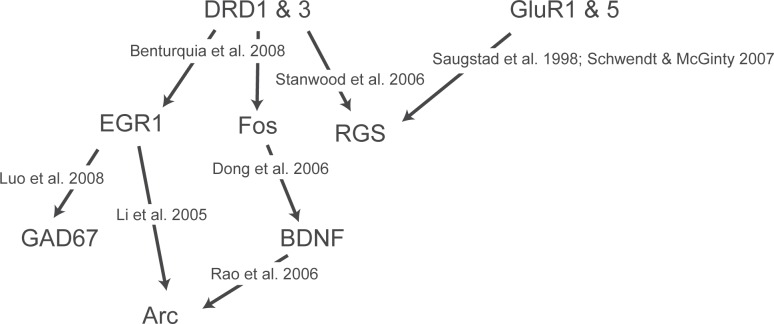
Genes discussed in this review can be placed into a hypothetical pathway of interaction. References depicted provide documentation of the intermolecular interactions. Clearly, the presence of all these relationships in a single cell is purely conjectural at this time.

AMPA receptors are composed of multiple glutamate receptor subunits, and expression of these glutamate receptor subunits is altered during withdrawal from cocaine (Lu et al. 2005) and heroin (Zhong et al. 2006; Bossert et al. 2006). Glutamic acid decarboxylase, isoform 67 (GAD67) is a GABA-synthesizing enzyme whose expression is known to be changed following drug use. After exposure to schedules of either morphine, amphetamine, or nicotine that induce behavioral sensitization, GAD67 mRNA expression is significantly increased in the central amygdala (Carta et al. 2008). This increase is evident following an initial drug treatment period and remains following sensitization, rather than a change specifically induced by the sensitization procedure. In hippocampal neurons, GAD67 gene expression is induced by EGR1 (Luo et al. 2008), suggesting a possible pathway through which some of the observed drug-induced changes in gene expression are coordinated (Fig. 4).

Additional genes whose expression is reportedly increased following abstinence from heroin and decreased following abstinence from cocaine are Arc (activity-regulated cytoskeletal protein) and c-fos. Studies of relapse to cocaine-seeking report EGR1, Arc, and c-fos gene expression to be increased following cocaine abstinence and contextual re-exposure (Klebaur et al. 2002; Ostrander et al. 2003; Zavala et al. 2008; Hearing et al. 2008b). Changes in Arc and c-fos gene expression also exist in the amygdala following re-exposure to an environment paired with opiate withdrawal, although the direction and magnitude of change is variable between sub-nuclei regions (Lucas et al. 2008). Comparing the basolateral amygdala (BLA), intercalated cell masses (ITC), and central nucleus of the amygdala (CeA) following re-exposure to a drug-paired environment, both Arc and c-fos increased more in cells that were also positive for GAD67 when examined in the ITC and CeA. However, in the BLA, expression of both genes was higher in GAD67 negative neurons.

Contextual cues play a crucial role in neuronal gene expression changes that are associated with drug use. Several immediate early genes are reportedly upregulated when rats are reintroduced to an environment in which they previously self-administered cocaine, and neuronal regions including the nucleus accumbens (Fuchs et al. 2008), dorsal striatum (See et al. 2007), prefrontal cortex, amygdala, and hippocampus (Fuchs et al. 2005). Thus, the context of drug-taking affects gene expression.

## Epigenetics

The field of epigenetics examines how intranuclear changes in the structure of DNA and chromatin can produce changes in gene transcription (Colvis et al. 2005). Changes in gene expression are the endpoint of complex interactions of histones and chromatin that determine which genes will be transcribed (Renthal and Nestler, 2008). Histone acetylation, histone methylation, histone phosphorylation, and DNA methylation combine to determine gene expression levels, and thus these are the mechanisms by which drugs actually elicit genomic effects. DNA is normally tightly wound around histones, forming the basis for chromatin structure. Transcription factors gain access to DNA that is not tightly wound around histones, and therefore epigenetic changes are central to determining which genes will be transcribed, and ultimately result in alterations in gene expression levels. In the nucleus accumbens, histone acetylation is induced by both acute and chronic cocaine administration (Kumar et al. 2005), although acute administration is associated with acetylation of H4 while chronic administration results in acetylation of H3. Acute administration results in deacetylation that promotes c-fos gene expression, while chronic administration accompanies histone deacetylation that promotes BDNF and cdk5 gene expression. In the mPFC, decreased levels of H3 aceytylation are seen both 1 and 100 days after cocaine self-administration (Freeman et al. 2008). In addition to the cocaine administration, cocaine-induced sensitization has also been associated with chromatin remodeling (Schroeder et al. 2008). In this case, changes in BDNF and D1 dopamine receptor gene expression were identified. Histone deacetylases remove acetyl groups from histones to change chromatin structure, and changes in expression of these enzymes could be responsible for drug-induced acetylation changes. Function of histone deacetylase 5 (HDAC5) is decreased in the NAc after chronic cocaine exposure (Renthal et al. 2007), and this decreased function alters the transcription pattern for several genes. Histone deacetylase 1 (HDAC1) affects c-fos gene expression following amphetamine exposure (Renthal et al. 2008). Expression of HDAC1 is normally chronically repressed by ΔfosB, but when it is expressed, HDAC1 allows chromatin remodeling that promotes c-fos gene expression.

Changes in mRNA levels that are correlated with changes in the chromatin modification of their respective genes have been documented. Thus, the role of chromatin structure in drug-induced changes in gene expression is a field which is rich in potential discoveries. Epigenetic studies are destined to provide a deeper understanding of the detailed mechanisms behind changes in gene expression.

## Gene Intervention Behavioral Studies

Although many genes can be identified as being changed following drug use and relapse, the relevance of these genes to relapse must be assessed through behavioral studies. Fortunately, technologies such as siRNA, antisense oligonucleotides, and genetic knockouts allow the importance of specific genes, in specific neuronal areas, to be assessed. Infusion of EGR1 antisense RNA into the amygdala 90 min prior to a session of memory reactivation abolishes cue-induced reinstatement of cue-induced cocaine-seeking (Lee et al. 2005; Lee et al. 2006). Because drug-seeking behavior was reduced when EGR1 gene expression was reduced immediately prior to reconsolidation, the role of EGR1 in relapse appears to be closely tied to memory formation. This finding suggests EGR1 is an important gene for relapse, but because EGR1 functions as a transcription factor, the changes in expression of several genes transcribed by EGR1 is likely important to relapse. In fact, this is the authors’ conclusion and points to the importance of further studies to gain a broad scope of genomic changes. Genetic manipulation, combined with behavioral relapse models, provides a powerful approach for investigating the relevance of particular genes to relapse. Intracranial infusions of antisense oligodeoxynucleotides (against EGR1) into rat amygdala prior to reactivation and reconsolidation of drug-seeking memory reduces seeking behavior for both cocaine and heroin (Hellemans et al. 2006; Lee et al. 2006). The gene-intervention studies discussed in this paper utilize antisense oligomers. However, oligos often have toxicity and it is necessary to confirm there is no damage to physiological functions or regions other than the one targeted. Therefore, models in which suppression of gene expression does not require introduction of a foreign agent are preferable for examining the role of specific genes.

## Future Directions

### Dynamic database

The studies discussed thus far provide a summary of what we currently know about genes whose expression levels are changed in models of relapse, but further steps must be taken to ensure a continuing increase in knowledge about relapse to drug abuse. An excellent start will be the development of a dynamic database to report genes that have been found to be implicated in relapse. A database similar to this was initially produced for cocaine-responsive genes (Freeman et al. 2002), but newer, web-based approaches, such as the Allen Brain Atlas (www.brain-map.org) could combine drug, behavioral, anatomical, and gene expression data. A central repository for relapse information will accelerate the progress that can be made in further experiments investigating relapse to drug use.

### Temporally and spatially-selective knock-outs

The relevance of specific genes to addiction can be studied using transgenic mice. Mice that are heterozygous or homozygous mutants for the EGR1 immediate early gene show behavioral differences from their wild type counterparts. Specifically, cocaine-induced locomotor sensitization is significantly lower in EGR1−/− and EGR1+/− mice than in EGR1+/+ mice (Valjent et al. 2006). To ensure that EGR1 was the only transcription factor affected by the knock-in, the researchers in this particular study examined pathways upstream from EGR1 and parallel signaling pathways. Because EGR1 is a transcription factor, a valid concern is the possibility that another (or several other) genes whose transcription is regulated by EGR1 are affected. An ideal experiment to examine the relevance of particular genes to relapse is to use mice in which gene knockouts are both temporally and spatially specific. Transgenic mice, such as CRE-LacZ reporter mice (Shaw-Lutchman et al. 2003), provide ideal models in which such studies can be conducted. Combining measurements of transcription with and without expression of a particular gene with drug exposure provides insight about whether a particular gene is affected by that drug (Green et al. 2006). This approach can be used in models of relapse to drug-seeking to establish the importance of particular genes to the relapse phenomenon. Of course, the difficulty in this approach is the paucity of relapse mouse models. Therefore, further developments in either murine models of relapse or rat genetic manipulation (such as viral delivery of shRNA) will be required before the power of this approach is fully harnessed. The importance of cAMP-response element (CRE) to opiate withdrawal behavior has been determined using a transgenic mouse model (Shaw-Lutchman et al. 2002; Han et al. 2006). Region-specific changes in CRE-mediated transcription occur during opiate withdrawal, and CRE activity increases opiate withdrawal while decreasing CRE activity decreases opiate withdrawal.

The importance of glutamate receptors to sensitization to cocaine is apparent through a study using viral-mediated gene transfer of wild-type or pore-dead Glutamate receptor 1 (GluR1) into rat NAc (Backtell et al. 2008). Infusions of wt-GluR1 viral vectors result in a decrease in cocaine sensitization. Cocaine-seeking during extinction and cocaine-induced reinstatement were also reduced in rats in which GluR1 was overexpressed. Further studies will be needed to elucidate how GluR1 overexpression prevents these relapse measures in animal models, but it is likely that many of the genes highlighted in this review are involved in the mechanism.

### Drug discovery

An ultimate goal of drug abuse research is to design pharmaceutical treatments to prevent relapse. Identification of target genes is the first step in drug discovery, and a database of genes suspected to be related to relapse could be valuable to making the drug discovery process more efficient. Now that research has determined that intervention with EGR1 gene expression decreases relapse, the search for pharmaceutical agents that affect EGR1 gene expression and downstream targets of EGR1 in specific neuronal regions should begin. Future relapse prevention research should occur on multiple levels, ranging from molecular to behavioral, and combining different fields as often as possible.

## Conclusion

Knowledge of the gene expression patterns that exist following prolonged drug use and abstinence is useful in understanding the neurobiological drive to relapse despite the ensuing adverse consequences. How does one progress from taking a drug because it elicits rewarding effects to engaging in drug use as a necessity? The answer to this question may lie in the intricate molecular changes occurring in neurons during drug use and abstinence. These molecular changes, however, will be intricately intertwined with learning and memory. Although the focus of this article has been on drug addiction, the contribution of some of these genes to relapse may be through their role in memory formation and retrieval. Multiple hypotheses exist to explain addiction (Robinson and Berridge, 2003; Thomas et al. 2008; Koob and Le Moal, 2008a; Koob and Le Moal, 2008b). Whether addiction and relapse stem from the avoidance of unpleasant physical states, motivational systems that drive compulsive behavior, or memories that refuse to be silenced, is a topic of debate, and components of each theory are likely necessary for a complete understanding of addiction. The genomic profile of former addicts who are highly susceptible to relapse represents a combination of memory and drug-induced changes, and knowledge of this pattern of gene expression will be critical for designing the most effective pharmacological treatments for relapse.

## Figures and Tables

**Table 1:** Several genes have been identified to be changed in models of relapse.

Gene	Full Name	Source
EGR1	Early growth response 1	Kuntz et al. 2008; Schmidt et al. 2005; Hellemans et al. 2006; Covington et al. 2005; Freeman et al. 2008; Lee et al. 2006
BDNF	Brain-derived neurotrophic factor	Graham et al. 2007; Pu et al. 2006; Liu et al. 2005, 2008; Filip et al. 2006; Itoh et al. 2005; Le et al. 2005
DRD2	Dopamine receptor D2	Li et al. 2006; Shao et al. 2006; David et al. 2007, 2008
DRD4	Dopamine receptor D4	Li et al. 2006; Shao et al. 2006; David et al. 2007, 2008
RGS2	Regulator of G-protein signaling 2	Gold et al. 2003; Stanwood et al. 2006
RGS4	Regulator of G-protein signaling 4	Gold et al. 2003; Schwendt et al. 2007; Stanwood et al. 2006
RGS9	Regulator of G-protein signaling 9	Zachariou et al. 2003; Stanwood et al. 2006
GluR1	Glutamate receptor subunit GluR1	Backtell et al. 2008
Arc	Activity regulated cytoskeletal-associated protein	Klebaur et al. 2002; Zavala et al. 2008; Hearing et al. 2008
c-fos	Fos	Ostramdar et al. 2003
GAD67	Glutamic acid decarboxylase	Carta et al. 2008

## References

[b1-sart-recentdevelopmentsinsubstanceabuseresearchandtreatmentspecialissue2008-2008-001] Abramow-Newerly M, Roy AA, Nunn C, Chidiac P (2006). RGS proteins have a signalling complex: interactions between RGS proteins and GPCRs, effectors, and auxiliary proteins. Cell Signal.

[b2-sart-recentdevelopmentsinsubstanceabuseresearchandtreatmentspecialissue2008-2008-001] Alcaro A, Huber R, Panksepp J (2007). Behavioral functions of the mesolimbic dopaminergic system: an affective neuroethological perspective. Brain Res Rev.

[b3-sart-recentdevelopmentsinsubstanceabuseresearchandtreatmentspecialissue2008-2008-001] Backtell RA, Choi KH, Simmons DL, Falcon E, Monteggia LM, Neve RL, Self DW (2008). Role of GluR1 expression in nucleus accumbens neurons in cocaine sensitization and cocaine-seeking behavior. Eur J Neurosci.

[b4-sart-recentdevelopmentsinsubstanceabuseresearchandtreatmentspecialissue2008-2008-001] Badiani A, Robinson TE (2004). Drug-induced neurobehavioral plasticity: the role of environmental context. Behav Pharmacol.

[b5-sart-recentdevelopmentsinsubstanceabuseresearchandtreatmentspecialissue2008-2008-001] Badiani A, Oates MM, Day HE, Watson SJ, Akil H, Robinson TE (1998). Amphetamine-induced behavior, dopamine release, and c-fos mRNA expression: modulation by environmental novelty. J Neurosci.

[b6-sart-recentdevelopmentsinsubstanceabuseresearchandtreatmentspecialissue2008-2008-001] Bossert JM, Gray SM, Lu L, Shaham Y (2006). Activation of group II metabotropic glutamate receptors in the nucleus accumbens shell attenuates context-induced relapse to heroin seeking. Neuropsychopharmacology.

[b7-sart-recentdevelopmentsinsubstanceabuseresearchandtreatmentspecialissue2008-2008-001] Carta AR, Moreno CC, Cadoni C, Tronci E, Di CG (2008). Long-term increase in GAD67 mRNA expression in the central amygdala of rats sensitized by drugs and stress. Eur J Neurosci.

[b8-sart-recentdevelopmentsinsubstanceabuseresearchandtreatmentspecialissue2008-2008-001] Colvis CM, Pollock JD, Goodman RH, Impey S, Dunn J, Mandel G, Champagne FA, Mayford M, Korzus E, Kumar A, Renthal W, Theobald DE, Nestler EJ (2005). Epigenetic mechanisms and gene networks in the nervous system. J Neurosci.

[b9-sart-recentdevelopmentsinsubstanceabuseresearchandtreatmentspecialissue2008-2008-001] Comings DE, Gade R, Wu S, Chiu C, Dietz G, Muhleman D, Saucier G, Ferry L, Rosenthal RJ, Lesieur HR, Rugle LJ, MacMurray P (1997). Studies of the potential role of the dopamine D1 receptor gene in addictive behaviors. Mol Psychiatry.

[b10-sart-recentdevelopmentsinsubstanceabuseresearchandtreatmentspecialissue2008-2008-001] Covington HE, Kikusui T, Goodhue J, Nikulina EM, Hammer RP, Miczek KA (2005). Brief social defeat stress: long lasting effects on cocaine taking during a binge and zif268 mRNA expression in the amygdala and prefrontal cortex. Neuropsychopharmacology.

[b11-sart-recentdevelopmentsinsubstanceabuseresearchandtreatmentspecialissue2008-2008-001] Crombag HS, Bossert JM, Koya E, Shaham Y (2008). Review. Context-induced relapse to drug seeking: a review. Philos Trans R Soc Lond B Biol Sci.

[b12-sart-recentdevelopmentsinsubstanceabuseresearchandtreatmentspecialissue2008-2008-001] David SP, Munafo MR, Murphy MF, Proctor M, Walton RT, Johnstone EC (2008). Genetic variation in the dopamine D4 receptor (DRD4) gene and smoking cessation: follow-up of a randomised clinical trial of transdermal nicotine patch. Pharmacogenomics J.

[b13-sart-recentdevelopmentsinsubstanceabuseresearchandtreatmentspecialissue2008-2008-001] David SP, Strong DR, Munafo MR, Brown RA, Lloyd-Richardson EE, Wileyto PE, Evins EA, Shields PG, Lerman C, Niaura R (2007). Bupropion efficacy for smoking cessation is influenced by the DRD2 Taq1A polymorphism: analysis of pooled data from two clinical trials. Nicotine Tob Res.

[b14-sart-recentdevelopmentsinsubstanceabuseresearchandtreatmentspecialissue2008-2008-001] DeJong W (1994). Relapse prevention: an emerging technology for promoting long-term drug abstinence. Int J Addict.

[b15-sart-recentdevelopmentsinsubstanceabuseresearchandtreatmentspecialissue2008-2008-001] DiChiara G, Bassareo V (2007). Reward system and addiction: what dopamine does and doesn’t do. Curr Opin Pharmacol.

[b16-sart-recentdevelopmentsinsubstanceabuseresearchandtreatmentspecialissue2008-2008-001] Epstein DH, Preston KL, Stewart J, Shaham Y (2006). Toward a model of drug relapse: an assessment of the validity of the reinstatement procedure. Psychopharmacology (Berl).

[b17-sart-recentdevelopmentsinsubstanceabuseresearchandtreatmentspecialissue2008-2008-001] Falcon E, McClung CA (2008). A role for the circadian genes in drug addiction. Neuropharmacology.

[b18-sart-recentdevelopmentsinsubstanceabuseresearchandtreatmentspecialissue2008-2008-001] Feldman RS, Meyer JS, Quenzer LF (1997). Principles of Neuropsychopharmacology.

[b19-sart-recentdevelopmentsinsubstanceabuseresearchandtreatmentspecialissue2008-2008-001] Ferguson SM, Norton CS, Watson SJ, Akil H, Robinson TE (2003). Amphetamine-evoked c-fos mRNA expression in the caudate-putamen: the effects of DA and NMDA receptor antagonists vary as a function of neuronal phenotype and environmental context. J Neurochem.

[b20-sart-recentdevelopmentsinsubstanceabuseresearchandtreatmentspecialissue2008-2008-001] Filip M, Faron-Gorecka A, Kusmider M, Golda A, Frankowska M, Dziedzicka-Wasylewska M (2006). Alterations in BDNF and trkB mRNAs following acute or sensitizing cocaine treatments and withdrawal. Brain Res.

[b21-sart-recentdevelopmentsinsubstanceabuseresearchandtreatmentspecialissue2008-2008-001] Freeman WM, Patel KM, Brucklacher RM, Lull ME, Erwin M, Morgan D, Roberts DC, Vrana KE (2008). Persistent Alterations in Mesolimbic Gene Expression with Abstinence from Cocaine Self-Administration. Neuropsychopharmacology.

[b22-sart-recentdevelopmentsinsubstanceabuseresearchandtreatmentspecialissue2008-2008-001] Freeman WM, Dougherty KE, Vacca SE, Vrana KE (2002). An interactive database of cocaine-responsive gene expression. Scientific World Journal.

[b23-sart-recentdevelopmentsinsubstanceabuseresearchandtreatmentspecialissue2008-2008-001] Fuchs RA, Ramierez DR, Bell GH (2008). Nucleus accumbens shell and core involvement in drug context-induced reinstatement of cocaine seeking in rats. Psychopharmacology (Berl).

[b24-sart-recentdevelopmentsinsubstanceabuseresearchandtreatmentspecialissue2008-2008-001] Fuchs RA, Evans KA, Ledford CC, Parker MP, Case JM, Mehta RH, See RE (2005). The role of the dorsomedial prefrontal cortex, basolateral amygdala, and dorsal hippocampus in contextual reinstatement of cocaine seeking in rats. Neuropsychopharmacology.

[b25-sart-recentdevelopmentsinsubstanceabuseresearchandtreatmentspecialissue2008-2008-001] Gold SJ, Han MH, Herman AE, Ni YG, Pudiak CM, Aghajanian GK, Liu RJ, Potts BW, Mumby SM, Nestler EJ (2003). Regulation of RGS proteins by chronic morphine in rat locus coeruleus. Eur J Neurosci.

[b26-sart-recentdevelopmentsinsubstanceabuseresearchandtreatmentspecialissue2008-2008-001] Graham DL, Edwards S, Bachtell RK, DiLeone RJ, Rios M, Self DW (2007). Dynamic BDNF activity in nucleus accumbens with cocaine use increases self-administration and relapse. Nat Neurosci.

[b27-sart-recentdevelopmentsinsubstanceabuseresearchandtreatmentspecialissue2008-2008-001] Green TA, Alibhai IN, Hommel JD, DiLeone RJ, Kumar A, Theobald DE, Neve RL, Nestler EJ (2006). Induction of inducible cAMP early repressor expression in nucleus accumbens by stress or amphetamine increases behavioral responses to emotional stimuli. J Neurosci.

[b28-sart-recentdevelopmentsinsubstanceabuseresearchandtreatmentspecialissue2008-2008-001] Grimm JW, Hope BT, Wise RA, Shaham Y (2001). Neuroadaptation. Incubation of cocaine craving after withdrawal. Nature.

[b29-sart-recentdevelopmentsinsubstanceabuseresearchandtreatmentspecialissue2008-2008-001] Guillin O, Diaz J, Carroll P, Griffon N, Schwartz JC, Sokoloff P (2001). BDNF controls dopamine D3 receptor expression and triggers behavioural sensitization. Nature.

[b30-sart-recentdevelopmentsinsubstanceabuseresearchandtreatmentspecialissue2008-2008-001] Guitart-Masip M, Johansson B, Canete T, Fernandez-Teruel A, Tobena A, Terenis L, Gimenez-Llort L (2008). Regional adaptations in PSD-95, NGFI-A and secretogranin gene transcripts related to vulnerability to behavioral sensitization to amphetamine in the Roman rat strains. Neuroscience.

[b31-sart-recentdevelopmentsinsubstanceabuseresearchandtreatmentspecialissue2008-2008-001] Han MH, Bolanos CA, Green TA, Olson VG, Neve RL, Liu RJ, Aghajanian GK, Nestler EJ (2006). Role of cAMP response element-binding protein in the rat locus ceruleus: regulation of neuronal activity and opiate withdrawal behaviors. J Neurosci.

[b32-sart-recentdevelopmentsinsubstanceabuseresearchandtreatmentspecialissue2008-2008-001] Hearing MC, Miller SW, See RE, McGinty JF (2008a). Relapse to cocaine seeking increases activity-regulated gene expression differentially in the prefrontal cortex of abstinent rats. Psychopharmacology (Berl).

[b33-sart-recentdevelopmentsinsubstanceabuseresearchandtreatmentspecialissue2008-2008-001] Hearing MC, See RE, McGinty JF (2008b). Relapse to cocaine-seeking increases activity-regulated gene expression differentially in the striatum and cerebral cortex of rats following short or long periods of abstinence. Brain Struct Funct.

[b34-sart-recentdevelopmentsinsubstanceabuseresearchandtreatmentspecialissue2008-2008-001] Hellemans KG, Everitt BJ, Lee JL (2006). Disrupting reconsolidation of conditioned withdrawal memories in the basolateral amygdala reduces suppression of heroin seeking in rats. J Neurosci.

[b35-sart-recentdevelopmentsinsubstanceabuseresearchandtreatmentspecialissue2008-2008-001] Hemby SE (2006). Assessment of genome and proteome profiles in cocaine abuse. Prog Brain Res.

[b36-sart-recentdevelopmentsinsubstanceabuseresearchandtreatmentspecialissue2008-2008-001] Itoh K, Hashimoto K, Shimizu E, Sekine Y, Ozaki N, Inada T, Harano M, Iwata N, Komiyama T, Yamada M, Sora I, Nakata K, Ujike H, Iyo M (2005). Association study between brain-derived neurotrophic factor gene polymorphisms and methamphetamine abusers in Japan. Am J Med Genet B Neuropsychiat Genet.

[b37-sart-recentdevelopmentsinsubstanceabuseresearchandtreatmentspecialissue2008-2008-001] Kalivas PW, Duffy P, White SR (1998a). MDMA elicits behavioral and neurochemical sensitization in rats. Neuropsychopharmacology.

[b38-sart-recentdevelopmentsinsubstanceabuseresearchandtreatmentspecialissue2008-2008-001] Kalivas PW, Pierce RC, Cornish J, Sorg BA (1998b). A role for sensitization in craving and relapse in cocaine addiction. J Psychopharmacol.

[b39-sart-recentdevelopmentsinsubstanceabuseresearchandtreatmentspecialissue2008-2008-001] Katz JL, Higgins ST (2003). The validity of the reinstatement model of craving and relapse to drug use. Psychopharmacology (Berl).

[b40-sart-recentdevelopmentsinsubstanceabuseresearchandtreatmentspecialissue2008-2008-001] Klebaur JE, Ostrander MM, Norton CS, Watson SJ, Akil H, Robinson TE (2002). The ability of amphetamine to evoke arc (Arg 3.1) mRNA expression in the caudate, nucleus accumbens and neocortex is modulated by environmental context. Brain Res.

[b41-sart-recentdevelopmentsinsubstanceabuseresearchandtreatmentspecialissue2008-2008-001] Koob G, Le Moal M (2008a). Addiction and the brain antireward system. Ann Rev Physiol.

[b42-sart-recentdevelopmentsinsubstanceabuseresearchandtreatmentspecialissue2008-2008-001] Koob G, Le Moal M (2008b). Neurobiological mechanisms for opponent motivational processes in addiction. Philos Trans R Soc Lond B Biol Sci.

[b43-sart-recentdevelopmentsinsubstanceabuseresearchandtreatmentspecialissue2008-2008-001] Kreek MJ, Bart G, Lilly C, LaForge KS, Nielsen DA (2005). Pharmacogenetics and human molecular genetics of opiate and cocaine addictions and their treatments. Pharmacol Rev.

[b44-sart-recentdevelopmentsinsubstanceabuseresearchandtreatmentspecialissue2008-2008-001] Krumins AM, Barker SA, Huang C, Sunahara RK, Yu K, Wilkie TM, Gold SJ, Mumby SM (2004). Differentially regulated expression of endogenous RGS4 and RGS7. J Biol Chem.

[b45-sart-recentdevelopmentsinsubstanceabuseresearchandtreatmentspecialissue2008-2008-001] Kumar A, Choi KH, Renthal W, Tsankova NM, Theobald DE, Truong HT, Russo SJ, Laplant Q, Sasaki TS, Whistler KN, Neve RL, Self DW, Nestler EJ (2005). Chromatin remodeling is a key mechanism underlying cocaine-induced plasticity in striatum. Neuron.

[b46-sart-recentdevelopmentsinsubstanceabuseresearchandtreatmentspecialissue2008-2008-001] Kuntz KL, Twining RC, Baldwin AE, Vrana KE, Grigson PS (2008a). Heroin self-administration: I. Incubation of goal-directed behavior in rats. Pharmacol Biochem Behav.

[b47-sart-recentdevelopmentsinsubstanceabuseresearchandtreatmentspecialissue2008-2008-001] Kuntz KL, Patel KM, Grigson PS, Freeman WM, Vrana KE (2008b). Heroin self-administration: II. CNS gene expression following withdrawal and cue-induced drug-seeking behavior. Pharmacol Biochem Behav.

[b48-sart-recentdevelopmentsinsubstanceabuseresearchandtreatmentspecialissue2008-2008-001] Le FB, Diaz J, Sokoloff P (2005). A single cocaine exposure increases BDNF and D3 receptor expression: implications for drug-conditioning. Neuroreport.

[b49-sart-recentdevelopmentsinsubstanceabuseresearchandtreatmentspecialissue2008-2008-001] Lee JL, Milton AL, Everitt BJ (2006). Cue-induced cocaine seeking and relapse are reduced by disruption of drug memory reconsolidation. J Neurosci.

[b50-sart-recentdevelopmentsinsubstanceabuseresearchandtreatmentspecialissue2008-2008-001] Lee JL, Di CP, Thomas KL, Everitt BJ (2005). Disrupting reconsolidation of drug memories reduces cocaine-seeking behavior. Neuron.

[b51-sart-recentdevelopmentsinsubstanceabuseresearchandtreatmentspecialissue2008-2008-001] Leshner AI (1996). Understanding drug addiction: implications for treatment. Hosp Pract (Minneap).

[b52-sart-recentdevelopmentsinsubstanceabuseresearchandtreatmentspecialissue2008-2008-001] Li Y, Shao C, Zhang D, Zhao M, Lin L, Yan P, Xie Y, Jiang K, Jin L (2006). The effect of dopamine D2, D5 receptor and transporter (SLC6A3) polymorphisms on the cue-elicited heroin craving in Chinese. Am J Med Genet B Neuropsychiatr Genet.

[b53-sart-recentdevelopmentsinsubstanceabuseresearchandtreatmentspecialissue2008-2008-001] Liu QR, Zhu XG, Gong JP, Shaham Y, Uhl GR (2008). Rodent BDNF genes, novel promoters, novel splice variants, and regulation by cocaine. Brain Res.

[b54-sart-recentdevelopmentsinsubstanceabuseresearchandtreatmentspecialissue2008-2008-001] Liu QR, Walther D, Drgon T, Polesskaya O, Lesnick TG, Strain KJ, de AM, Bower JH, Maraganore DM, Uhl GR (2005). Human brain derived neurotrophic factor (BDNF) genes, splicing patterns, and assessments of associations with substance abuse and Parkinson’s Disease. Am J Med Genet B Neuropsychiatr Genet.

[b55-sart-recentdevelopmentsinsubstanceabuseresearchandtreatmentspecialissue2008-2008-001] Lu L, Koya E, Zhai H, Hope BT, Shaham Y (2006). Role of ERK in cocaine addiction. Trends Neurosci.

[b56-sart-recentdevelopmentsinsubstanceabuseresearchandtreatmentspecialissue2008-2008-001] Lu L, Dempsey J, Shaham Y, Hope BT (2005). Differential long-term neuroadaptations of glutamate receptors in the basolateral and central amygdala after withdrawal from cocaine self-administration in rats. J Neurochem.

[b57-sart-recentdevelopmentsinsubstanceabuseresearchandtreatmentspecialissue2008-2008-001] Lucas M, Frenois F, Vouillac C, Stinus L, Cador M, Le MC (2008). Reactivity and plasticity in the amygdala nuclei during opiate withdrawal conditioning: Differential expression of c-fos and arc immediate early genes. Neuroscience.

[b58-sart-recentdevelopmentsinsubstanceabuseresearchandtreatmentspecialissue2008-2008-001] Luo Y, Lathia J, Mughal M, Mattson MP (2008). SDF1alpha/CXCR.4 signaling, Via ERKs and the transcription factor Egr1, induces expression of a 67-kDa form of glutamic acid decarboxylase in embryonic hippocampal neurons. J Biol Chem.

[b59-sart-recentdevelopmentsinsubstanceabuseresearchandtreatmentspecialissue2008-2008-001] McClung CA, Nestler EJ (2003). Regulation of gene expression and cocaine reward by CREB. and DeltaFosB. Nat Neurosci.

[b60-sart-recentdevelopmentsinsubstanceabuseresearchandtreatmentspecialissue2008-2008-001] Nestler EJ (2001). Molecular basis of long-term plasticity underlying addiction. Nat Rev Neurosci.

[b61-sart-recentdevelopmentsinsubstanceabuseresearchandtreatmentspecialissue2008-2008-001] Nestler EJ, Barrot M, Self DW (2001). DeltaFosB: a sustained molecular switch for addiction. Proc Natl Acad Sci USA.

[b62-sart-recentdevelopmentsinsubstanceabuseresearchandtreatmentspecialissue2008-2008-001] O’Brien CP (2004). The mosaic of addiction. Am J Psychiatry.

[b63-sart-recentdevelopmentsinsubstanceabuseresearchandtreatmentspecialissue2008-2008-001] O’Brien CP (2003). Research advances in the understanding and treatment of addiction. Am J Addict.

[b64-sart-recentdevelopmentsinsubstanceabuseresearchandtreatmentspecialissue2008-2008-001] Ostrander MM, Badiani A, Day HE, Norton CS, Watson SJ, Akil H, Robinson TE (2003). Environmental context and drug history modulate amphetamine-induced c-fos mRNA expression in the basal ganglia, central extended amygdala, and associated limbic forebrain. Neuroscience.

[b65-sart-recentdevelopmentsinsubstanceabuseresearchandtreatmentspecialissue2008-2008-001] Pu L, Liu QS, Poo MM (2006). BDNF-dependent synaptic sensitization in midbrain dopamine neurons after cocaine withdrawal. Nat Neurosci.

[b66-sart-recentdevelopmentsinsubstanceabuseresearchandtreatmentspecialissue2008-2008-001] Renthal W, Maze I, Vaishnav K, Covington HE, Xiao G, Kuman A, Russo SJ, Graham A, Tsankova N, Kippin TE, Kerstetter KA, Neve RL, Haggarty SJ, McKinsey TA, Bassel-Duby R, Olson EN, Nestler EJ (2007). Histone Deacetylase 5 epigenetically controls behavioral adaptations to chronic emotional stimuli. Neuron.

[b67-sart-recentdevelopmentsinsubstanceabuseresearchandtreatmentspecialissue2008-2008-001] Renthal W, Carle TL, Maze I, Covington HE, Truong HT, Alibhai I, Kumar A, Montgomery RL, Olson EN, Nestler EJ (2008). DeltaFosB. mediates epigenetic desensitization of the c-fos gene after chronic amphetamine exposure. J Neurosci.

[b68-sart-recentdevelopmentsinsubstanceabuseresearchandtreatmentspecialissue2008-2008-001] Renthal W, Nestler EJ (2008). Epigenetic mechanisms in drug addiction. Trends Mol Med.

[b69-sart-recentdevelopmentsinsubstanceabuseresearchandtreatmentspecialissue2008-2008-001] Rogers JL, Ghee S, See RE (2008). The neural circuitry underlying reinstatement of heroin-seeking behavior in an animal model of relapse. Neuroscience.

[b70-sart-recentdevelopmentsinsubstanceabuseresearchandtreatmentspecialissue2008-2008-001] Schmidt ED, Voorn P, Binnekade R, Schoffelmeer AN, de Vries TJ (2005). Differential involvement of the prelimbic cortex and striatum in conditioned heroin and sucrose seeking following long-term extinction. Eur J Neurosci.

[b71-sart-recentdevelopmentsinsubstanceabuseresearchandtreatmentspecialissue2008-2008-001] Schroeder FA, Penta KL, Matevossian A, Jones SR, Konradi C, Tapper AR, Akbarian S (2008). Drug-Induced Activation of Dopamine D(1) Receptor Signaling and Inhibition of Class I/II Histone Deacetylase Induce Chromatin Remodeling in Reward Circuitry and Modulate Cocaine-Related Behaviors. Neuropsychopharmacology.

[b72-sart-recentdevelopmentsinsubstanceabuseresearchandtreatmentspecialissue2008-2008-001] Schwendt M, Hearing MC, See RE, McGinty JF (2007). Chronic cocaine reduces RGS4 mRNA in rat prefrontal cortex and dorsal striatum. Neuroreport.

[b73-sart-recentdevelopmentsinsubstanceabuseresearchandtreatmentspecialissue2008-2008-001] Schwendt M, Gold SJ, McGinty JF (2006). Acute amphetamine down-regulates RGS4 mRNA and protein expression in rat forebrain: distinct roles of D1 and D2 dopamine receptors. J Neurochem.

[b74-sart-recentdevelopmentsinsubstanceabuseresearchandtreatmentspecialissue2008-2008-001] See RA, Elliott JC, Feltenstein MW (2007). The role of dorsal vs ventral striatal pathways in cocaine-seeking behavior after prolonged abstinence in rats. Psychopharmacology (Berl).

[b75-sart-recentdevelopmentsinsubstanceabuseresearchandtreatmentspecialissue2008-2008-001] Shalev U, Morales M, Hope B, Yap J, Shaham Y (2001). Time-dependent changes in extinction behavior and stress-induced reinstatement of drug seeking following withdrawal from heroin in rats. Psychopharmacology (Berl).

[b76-sart-recentdevelopmentsinsubstanceabuseresearchandtreatmentspecialissue2008-2008-001] Shao C, Li Y, Jiang K, Zhang D, Xu Y, Lin L, Wang Q, Zhao M, Jin L (2006). Dopamine D4 receptor polymorphism modulates cue-elicited heroin craving in Chinese. Psychopharmacology (Berl).

[b77-sart-recentdevelopmentsinsubstanceabuseresearchandtreatmentspecialissue2008-2008-001] Shaw-Lutchman TZ, Impey S, Storm D, Nestler EJ (2003). Regulation of CRE-mediated transcription in mouse brain by amphetamine. Synapse.

[b78-sart-recentdevelopmentsinsubstanceabuseresearchandtreatmentspecialissue2008-2008-001] Shaw-Lutchman TZ, Barrot M, Wallace T, Gilden L, Zachariou V, Impey S, Duman RS, Storm D, Nestler EJ (2002). Regional and cellular mapping of cAMP response element-mediated transcription during naltrexone-precipitated morphine withdrawal. J Neurosci.

[b79-sart-recentdevelopmentsinsubstanceabuseresearchandtreatmentspecialissue2008-2008-001] Shepard JD, Bossert JM, Liu SY, Shaham Y (2004). The anxiogenic drug yohimbine reinstates methamphetamine seeking in a rat model of drug relapse. Biol Psychiatry.

[b80-sart-recentdevelopmentsinsubstanceabuseresearchandtreatmentspecialissue2008-2008-001] Smith MA, Ward SJ, Roberts DC (2008). Lesions of the dorsomedial frontal cortex block sensitization to the positive-reinforcing effects of cocaine. Pharmacol Biochem Behavior.

[b81-sart-recentdevelopmentsinsubstanceabuseresearchandtreatmentspecialissue2008-2008-001] Stanwood GD, Parlaman JP, Levitt P (2006). Genetic or pharmacological inactivation of the dopamine D1 receptor differentially alters the expression of regulator of G-protein signalling (Rgs) transcripts. Eur J Neurosci.

[b82-sart-recentdevelopmentsinsubstanceabuseresearchandtreatmentspecialissue2008-2008-001] Stewart J (2008). Psychological and neural mechanisms of relapse. Philos Trans R Soc Lond B Biol Sci.

[b83-sart-recentdevelopmentsinsubstanceabuseresearchandtreatmentspecialissue2008-2008-001] Thanos PK, Rivera SN, Weaver K, Grandy DK, Rubinstein M, Umegaki H, Wang GJ, Hitzemann R, Volkow ND (2005). Dopamine D2R. DNA transfer in dopamine D2 receptor-deficient mice: effects on ethanol drinking. Life Sci.

[b84-sart-recentdevelopmentsinsubstanceabuseresearchandtreatmentspecialissue2008-2008-001] Thanos PK, Taintor NB, Rivera SN, Umegaki H, Ikari H, Roth G, Ingram DK, Hitzemann R, Fowler JS, Gatley SJ, Wang GJ, Volkow ND (2004). DRD2 gene transfer into the nucleus accumbens core of the alcohol preferring and nonpreferring rats attenuates alcohol drinking. Alcohol Clin Exp Res.

[b85-sart-recentdevelopmentsinsubstanceabuseresearchandtreatmentspecialissue2008-2008-001] Thomas MJ, Kalivas PW, Shaham Y (2008). Neuroplasticity in the mesolimbic dopamine system and cocaine addiction. Br J Pharmacol.

[b86-sart-recentdevelopmentsinsubstanceabuseresearchandtreatmentspecialissue2008-2008-001] Valjent E, Aubier B, Corbille AG, Brami-Cherrier K, Caboche J, Topilko P, Girault JA, Herve D (2006). Plasticity-associated gene Krox24/Zif268 is required for long-lasting behavioral effects of cocaine. J Neurosci.

[b87-sart-recentdevelopmentsinsubstanceabuseresearchandtreatmentspecialissue2008-2008-001] Vezina P (2007). Sensitization, drug addiction and psychopathology in animals and humans. Prog Neuropsychopharmacol Biol Psychiatry.

[b88-sart-recentdevelopmentsinsubstanceabuseresearchandtreatmentspecialissue2008-2008-001] Worst TJ, Tan JC, Robertson DJ, Freeman WM, Hyytia P, Kiianmaa K, Vrana KE (2005). Transcriptome analysis of frontal cortex in alcohol-preferring and nonpreferring rats. J Neurosci Res.

[b89-sart-recentdevelopmentsinsubstanceabuseresearchandtreatmentspecialissue2008-2008-001] Xie GX, Palmer PP (2005). RGS proteins: new players in the field of opioid signaling and tolerance mechanisms. Anesth Analg.

[b90-sart-recentdevelopmentsinsubstanceabuseresearchandtreatmentspecialissue2008-2008-001] Yano M, Steiner H (2007). Methylphenidate and cocaine: the same effects on gene regulation?. Trends Pharmacol Sci.

[b91-sart-recentdevelopmentsinsubstanceabuseresearchandtreatmentspecialissue2008-2008-001] Zachariou V, Georgescu D, Sanchez N, Rahman Z, DiLeone R, Berton O, Neve RL, Sim-Selley LJ, Selley DE, Gold SJ, Nestler EJ (2003). Essential role for RGS9 in opiate action. Proc Natl Acad Sci USA.

[b92-sart-recentdevelopmentsinsubstanceabuseresearchandtreatmentspecialissue2008-2008-001] Zavala AR, Osredkar T, Joyce JN, Neisewander JL (2008). Upregulation of Arc mRNA expression in the prefrontal cortex following cue-induced reinstatement of extinguished cocaine-seeking behavior. Synapse.

[b93-sart-recentdevelopmentsinsubstanceabuseresearchandtreatmentspecialissue2008-2008-001] Zhang D, Zhang L, Tang Y, Zhang Q, Lao D, Sharp FR, Zhang J, Xu M (2005). Repeated cocaine administration induces gene expression changes through the dopamine D1 receptors. Neuropsychpharmacology.

[b94-sart-recentdevelopmentsinsubstanceabuseresearchandtreatmentspecialissue2008-2008-001] Zhong W, Dong Z, Tian M, Cao J, Xu T, Xu L, Luo J (2006). Opiate withdrawal induces dynamic expressions of AMPA receptors and its regulatory molecule CaMKIIalpha in hippocampal synapses. Life Sci.

